# Variation of mortality after coronary artery bypass surgery in relation to hour, day and month of the procedure

**DOI:** 10.1186/1471-2261-11-63

**Published:** 2011-10-20

**Authors:** Ann Coumbe, Ranjit John, Michael Kuskowski, Mehmet Agirbasli, Edward O McFalls, Selcuk Adabag

**Affiliations:** 1 Division of Cardiology, Veterans Administration Medical Center, One Veterans Drive, Minneapolis, Minnesota, 55417, USA; 2 Division of Cardiovascular Surgery, Veterans Administration Medical Center, One Veterans Drive, Minneapolis, Minnesota, 55417, USA; 3 Geriatric Research Education Center, Veterans Administration Medical Center, One Veterans Drive, Minneapolis, Minnesota, 55417, USA; 4 Department of Cardiology, Marmara University, Tophanelioglu caddesi, Altunizade, Istanbul, Turkey

**Keywords:** coronary artery bypass surgery, mortality

## Abstract

**Background:**

Mortality and complications after percutaneous coronary intervention is higher when performed after regular duty hours due to challenging patient characteristics, inferior processes of care and limited resources. Since these challenges are also encountered during coronary artery bypass graft (CABG) surgery that is performed after regular work hours, we assessed whether hour and day of procedure influenced mortality after CABG.

**Methods:**

We studied 4,714 consecutive patients who underwent CABG at the Minneapolis Veterans Administration (VA) Medical Center between 1987 and 2009. We compared postoperative (30-day) mortality rates in relation to hour and day in which the operation was performed.

**Results:**

Operations performed on weekends and after 4 PM had higher risk patients (p < 0.0001) and were more likely to be emergent (p < 0.0001), require intra-aortic balloon pump support (p < 0.0001) and result in postoperative complications (p < 0.0001) compared to those at regular work hours. Mortality was significantly higher when CABG was performed on weekends compared to weekdays (9.4% versus 2.5%; odds ratio (OR) 4.1, 95% confidence interval (CI) 1.6 to 10.4, p = 0.003), and after 4 PM compared to between 7 AM-4 PM (6.2% versus 2.2%; OR 2.9, 95% CI 1 to 8, p = 0.049). In multivariable analysis, when adjusted for the urgency of the operation and the VA estimated mortality risk score, these associations were no longer statistically significant.

**Conclusions:**

Mortality after CABG is higher when surgery is performed on the weekends and after 4 PM. These variations in mortality were related to higher patient risk, and urgency of the operation rather than external factors.

## Background

Coronary artery bypass graft (CABG) surgery and percutaneous coronary interventions (PCI) are performed at off-hours when the circumstances dictate to do so. It has been known that primary PCI, for acute myocardial infarction, have higher mortality and worse outcomes when performed in the evening hours or at night in comparison to day time [1-6]. This is thought to be secondary to challenging patient factors, such as hemodynamic instability, myocardial loss and more difficult lesion characteristics and inferior processes of care, such as longer door-to-balloon times, less supervision, operator fatigue and limited resources [1,2]. Thus, the results of PCI are less optimal in patients who present after regular work hours [1,2].

Published literature on the outcomes of surgery in relation to timing of procedure is incomplete. Previously, patients who underwent elective general surgery and peripheral vascular surgery in the evening were found to have a higher incidence of postoperative morbidity but not mortality [7]. Little is known about whether mortality after cardiac surgery varies with the time of the day, day of the week or the month of the year in which procedure was performed [8,9]. Further, potential factors that might influence such a variation in mortality are also unknown. Thus, the purpose of the present investigation was to examine the variation of mortality in relation to hour, day and month of procedure in a large cohort of patients who underwent CABG surgery at an academic center. We also aimed to examine the underlying patient and procedural factors that could contribute to such a variation. We hypothesized that the mortality after CABG is higher when it is performed after general work hours, akin to that observed in PCI, and in summer months when new trainees arrive.

## Methods

This study was approved by the Institutional Review Board and the Research and Development Committees of the Minneapolis Veterans Administration (VA) Medical Center. The individual consent was waived.

Minneapolis VA Medical Center is a tertiary referral center for VA facilities in the upper Midwest Integrated Service Network. Since 1987, almost 7000 cardiac surgeries (CABG and valve) were performed at our facility. More than 90% of the cardiac surgeries were performed by a stable cadre of 5 attending surgeons. Fellows in the University of Minnesota cardiothoracic surgery training program also perform operations at our facility under close supervision of the attending surgeon.

### Patient Selection and Data Collection

Of the 7000 cardiac surgical procedures performed at the Minneapolis VA Medical Center from June 1987 to March 2009, patients who underwent valve surgery alone or CABG-valve surgery were excluded. A total of 4714 consecutive patients who underwent CABG surgery alone were included in the present study. Baseline demographic and clinical data as well as operative details and outcomes were obtained from the cardiovascular surgery database at our medical center, which is an ongoing database of prospectively-collected data as part of a national database of all patients undergoing cardiac surgery at VA medical centers [10]. The database includes a validated mortality risk estimate (i.e. predicted probability of operative mortality) for each patient, based on his/her preoperative risk factors. The VA mortality risk estimate is obtained by stepwise logistic regression analysis of the variables entered into the national VA Continuous Improvement in Cardiac Surgery Program database. However, the date and time of the surgery is *not *included in the model. The VA mortality risk estimate has been previously validated as a predictor of postoperative mortality after cardiac surgery and shown to be superior to the Euroscore among VA patients [10-13].

The date of the cardiac surgery was recorded in all 4714 patients. In 2689 consecutive patients operated after October 1997 the starting time of the operation was also recorded. The priority of the surgery, as ascertained by the attending surgeon, was coded as elective, urgent or emergent.

### Outcome

The primary outcome of the study was operative mortality, defined as death within 30 days of the operation due to any cause or death after 30 days that is directly related to a complication of the cardiac surgery (e.g. mediastinitis).

### Statistical Analysis

Continuous variables are presented as mean ± standard deviation and categorical variables as percentages. Operative mortality rates and other categorical variables were compared using chi-square test. Continuous variables were compared with *t*-test. Logistic regression was used to obtain the odds ratios and 95% confidence intervals. Multivariable logistic regression included the time unit (day vs. night or weekday vs. weekend), mortality risk estimate and priority of the operation (elective vs. urgent or emergent). The method of adjusting for patients' risk by including the mortality risk estimate in the multivariable model has been validated before [14-16]. Indicator variables were used when examining the mortality rate in relation to the priority surgery and to the months of the year. Receiver Operating Characteristic curve was generated to assess the discriminative power of the mortality risk estimate. The area under the curve was displayed as the c-index. P value < 0.05 was taken as statistically significant. All analyses were performed SPSS version 19 statistical software.

## Results

### Patient Characteristics

Patients were 65 ± 9 years-old (range 34 to 87) and 99% were male (Table 1). Of the 4,714 CABG procedures, 3736 (79%) were elective, 662 (14%) were urgent and 308 (7%) were emergent. A total of 53 patients were operated on the weekend and 65 operated after 4 PM. Patients operated on weekends and after 4 PM were older, more likely to have prior myocardial infarction, more likely to undergo urgent/emergent surgery and require hemodynamic support with an intra-aortic balloon pump prior to surgery (Table 1). Consequently, these patients had higher operative risk based on higher American Society of Anesthesiologists score and VA mortality risk estimate in comparison to patients operated during weekdays and general work hours (Table 1).

**Table 1:** Baseline clinical characteristics of the 4714 study patients in relation to timing of coronary artery bypass surgery

	All patients n = 4714	Weekday n = 4661	Weekend n = 53	p value weekday vs. weekend	7 am-4 pm n = 2624	After 4 pm n = 65	p value 7 am-4 pm vs. after 4 pm
Age (years)	65 ± 9	65 ± 10	68 ± 11	0.015	65 ± 9	65 ± 10	0.96
Male	99%	99%	96%	0.06	99%	100%	1.0
Current smoker	22%	22%	30%	0.17	23%	30%	0.21
Diabetes mellitus	33%	33%	37%	0.53	37%	39%	0.82
Prior MI	53%	53%	73%	0.005	50%	59%	0.18
Prior heart surgery	8%	8%	11%	0.44	6%	3%	0.59
Intra-aortic balloon pump	3%	3%	32%	< 0.0001	3%	29%	< 0.0001
ASA score	3.2 ± 0.4	3.2 ± 0.4	3.6 ± 0.6	< 0.0001	3.2 ± 0.4	3.4 ± 0.5	0.009
Mortality estimate	3.6 ± 4	3.5 ± 4	11.4 ± 13	< 0.0001	3.3 ± 4	5.9 ± 6	0.001
Hemoglobin	13.7 ± 1.7	13.7 ± 1.7	11.9 ± 2.3	< 0.0001	13.7 ± 1.7	13.7 ± 1.8	0.93
Creatinine	1.3 ± 0.7	1.3 ± 0.7	1.3 ± 0.7	0.87	1.3 ± 0.7	1.3 ± 0.6	0.79
Albumin	4.1 ± 0.5	4.1 ± 0.5	3.7 ± 0.7	0.06	4.1 ± 0.5	3.9 ± 0.5	0.10

Abbreviations: ASA = American Society of Anesthesiologists; MI = myocardial infarction

### Predictors of Mortality

The expected mortality rate was 3.6 ± 4.5% (interquartile range 1.3% to 4.1%). The observed operative mortality rate was 2.6% (n = 121). Thus, the observed/expected mortality ratio was 0.72. Mortality estimate was a strong predictor of observed mortality (p < 0.0001) with a c-index 0.79 (95% confidence interval 0.74 to 0.83; p < 0.0001). The priority of CABG was also a predictor of mortality. In comparison to elective surgery, mortality rate was 1.6 times higher (95% confidence interval 0.99 to 2.65; p = 0.054) when CABG was urgent and 4.5 times higher (95% confidence interval 2.87 to 7.25; p < 0.0001) when emergent.

### Timing of CABG and Outcomes

Operations performed on the weekends and after 4 PM had similar cardiopulmonary bypass and ischemic times and similar number of grafts but significantly higher complication rates in comparison to those performed during general work hours (Table 2). Indeed, patients operated after regular work hours had a greater incidence of stroke, death and reoperation for bleeding and spent longer time on the ventilator postoperatively (Table 2).

**Table 2:** Details and complications of 4714 coronary artery bypass procedures in relation to timing of surgery

	All patients n = 4714	Weekday n = 4661	Weekend n = 53	p value weekday vs. weekend	7 am-4 pm n = 2624	After 4 pm n = 65	p value 7 am-4 pm vs. after 4 pm
Urgent/emergent surgery	21%	20%	91%	< 0.0001	15%	68%	< 0.0001
Number grafts	3 ± 1	3 ± 1	3 ± 1	0.15	3.1 ± 0.9	2.9 ± 1	0.18
bypass time (min)	123 ± 45	123 ± 45	111 ± 34	0.16	123 ± 45	112 ± 47	0.055
ischemic time (min)	80 ± 30	80 ± 30	73 ± 24	0.25	80 ± 30	74 ± 33	0.14
Operative mortality	2.6%	2.5%	9.4%	0.01	2.2%	6.2%	0.04
Stroke	1.6%	1.6%	1.9%	0.59	1.6%	6%	0.02
Reoperation for bleeding	3%	3%	4%	0.66	2%	11%	< 0.0001
Ventilated > 48 hours	7%	7%	19%	0.001	7%	20%	< 0.0001
Any complication	12%	12%	28%	< 0.0001	13%	28%	< 0.0001

**Figure 1:**
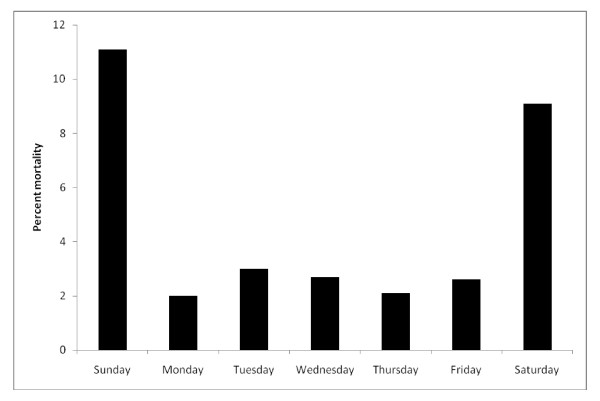
**Distribution of mortality in relation to the day of CABG surgery**.

The distribution of mortality rate in relation to the day of the week that the surgery was performed is demonstrated in Figure 1. Surgical operations performed on the weekend were associated with a 4-fold higher mortality than those performed during weekdays (9.4% versus 2.5%; odds ratio 4.1, 95% confidence interval 1.6 to 10.4, p = 0.003). In multivariable regression analysis, after adjusting for the priority of surgery and the mortality risk estimate, weekend surgery was no longer associated with increased mortality (odds ratio 1.18, 95% confidence interval 0.36 to 3.91; p = 0.78)

**Figure 2:**
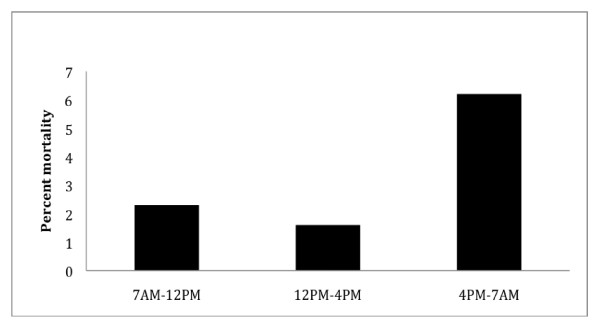
**Distribution of mortality in relation to the time of CABG surgery**.

The distribution of mortality rate in relation to the start time of the operation is demonstrated in Figure 2. Surgical procedures that started after 4 PM were associated with a 3-fold higher mortality than those started between 7 AM and 4 PM (6.2% versus 2.2%; odds ratio 2.9, 95% confidence interval 1 to 8, p = 0.049). In multivariable regression analysis, after adjusting for the priority of surgery and the mortality risk estimate, evening start time was no longer associated with increased mortality (odds ratio 2.2, 95% confidence interval 0.7 to7.1; p = 0.18).

**Figure 3:**
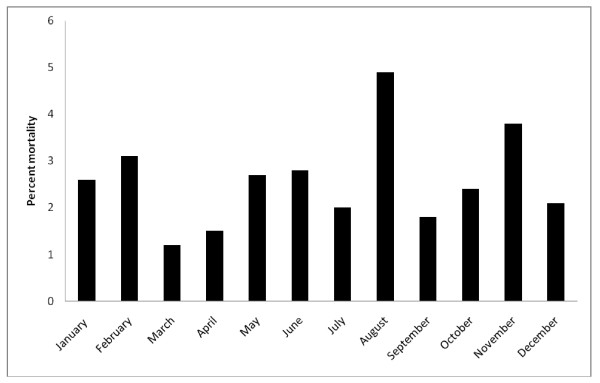
**Distribution of mortality in relation to the month of CABG surgery**. The monthly variation in mortality did not reach statistical significance (p = 0.08).

The distribution of mortality rate in relation to the months of the year is demonstrated in Figure 3. The mortality rate was highest in August (4.9%). However, the monthly variation in mortality did not reach statistical significance (p = 0.08). There was no difference in mortality with the seasons of the year (data not shown). Results did not change when off-pump surgeries were excluded from the analysis.

### Additional Analyses

Over the study period there were 308 emergency operations. Of these, 30 (10%) were operated during the weekend and 35 (11%) after 4 PM. The mortality rates of emergency surgical procedures performed during the weekday vs. weekend and between 7 AM-4 PM vs. after 4 PM were not significantly different (Table 3).

**Table 3:** Mortality rates in 308 emergency coronary artery bypass procedures in relation to timing of surgery

	All patients n = 308	Weekday n = 278	Weekend n = 30	p value weekday vs. weekend	7 am-4 pm n = 273	After 4 pm n = 35	p value 7 am-4 pm vs. after 4 pm
Operative mortality	8.4%	8.3%	10%	0.73	8.1%	11.4%	0.52

## Discussion

This investigation showed that cardiac surgical procedures that were performed after 4 PM and those that were performed on the weekends had 3 to 4 times higher mortality rate and increased complications when compared to CABG performed during the day and on weekdays, respectively. Patients who presented after regular work hours had higher surgical risk, were more likely to require urgent/emergent operation and need intra-aortic balloon pump prior to surgery to support their hemodynamic status. The incidence of reoperation for bleeding, postoperative stroke and prolonged ventilation was higher in these patients. When adjusted for the urgency of the operation and patient characteristics, timing-related variations in mortality disappeared. Previously, Dhadwal et al. [8] examined circadian variation of mortality in approximately 3000 patients undergoing CABG and found no relation between surgery start time and 30-day mortality in elective and urgent cases. However among patients who had emergency surgery, mortality rates were substantially higher in the morning and at night (~25% for both) compared to those operated in the afternoon (~3%). There was no biological or procedure-related explanation for this observation, however, and the authors cautioned that it might be a statistical aberration. Further, Tan et al. [9] studied patients who underwent elective CABG. There were no weekend cases and rare cases operated after 5 PM. They also found that operation start time, day, month and moon phase were not associated with outcome. Our study advances existing research in several respects. Neither of these previous studies was able to control for the patient risk factors as was done in the present study by adjusting for the VA mortality risk estimate. Further, procedural factors that likely influenced the outcome were not separated, as was done in the present study. Finally, there were no cases performed on the weekend [9]. Cumulative evidence from the present and previous studies suggests that patient's risk, urgency of the operation and suboptimal results dictate the outcome after cardiac surgery rather than circadian variation in the biological systems.

Time-related variation in mortality after non-cardiac surgery also appears to be related to patient factors. Kelz et al. examined 144,000 patients who underwent elective general surgery and peripheral vascular surgery [7]. In unadjusted analysis mortality was elevated in patients operated after 4 PM. However, after adjustment for patient risk factors start time of the surgery was not found to be associated with mortality any longer.

Outcomes after PCI for acute myocardial infarction are worse when PCI is performed after regular working hours [1-6]. Indeed, PCI failure rates and mortality were higher in patients who underwent procedures in the evening or at night after adjusting for baseline characteristics except in one relatively-small study of approximately 250 patients in which the mortality difference disappeared after adjusting for the CADILLAC risk score [5]. Magid et al. [1] reviewed approximately 30,000 patients who had primary PCI for acute myocardial infarction. They found that procedures performed after regular work hours had longer door-to-balloon time and higher mortality than those performed during regular work hours. Further, Glaser et al. [2] examined the potential causes for the increased mortality in approximately 700 patients who underwent primary PCI for myocardial infarction. Patients who had primary PCI after 7 PM were more likely to be smokers and more likely to present with cardiogenic shock. They had a higher incidence of multivessel coronary heart disease and a lower TIMI flow score. Patients who had PCI after 7 PM also had lower success rate in PCI and higher incidence of complications including coronary dissection [2]. Consequently, their rate of hospital mortality and recurrent myocardial infarction were also higher. After adjustment of the known patient factors outcomes after 7 PM were still inferior compared to day time.

Circadian variation of myocardial infarction, sudden cardiac death has been well-established [17-21]. Also, circadian variation has been demonstrated in the efficacy of thrombolytic therapy administered in the setting of acute ST-elevation myocardial infarction [22,23]. The mechanisms of these variations were thought to include diurnal variation in the activity of platelets and plasminogen activator-inhibitor-1 [22]. Indeed, plasminogen activator-inhibitor-1, a 47 kD single chain glycoprotein, is the main physiological inhibitor of tissue plasminogen activator and urokinase. Plasma PAI-1 levels show a circadian variation and peak in the early morning hours, coincident with the incidence of acute coronary events [24]. However, it is likely that patient factors and challenges in processes of care are responsible from the associations we have observed rather than circadian variation in biological factors.

The influence of training residents and fellows in an academic setting on surgical outcomes has been well documented. Periods of major turnover (beginning and end of surgical rotations) and the level of surgical resident training may particularly impact outcomes [25]. Shuhaiber et al. [26] determined that periods of major change in surgical staff are associated with increased risk-adjusted mortality after complex cardiac operations but not after CABG alone. Bakaeen et al. [27] found that while the early part of the academic year was associated with increased operative times, there were no differences in risk adjusted outcomes. Our results are consistent with these studies.

The management of coronary heart disease, acute coronary syndromes in particular, has changed significantly over time. The percutaneous interventional techniques are much more widely, and aggressively, employed in emergency cases than before. Many major medical centers have 24-hour cardiac catheterization laboratory coverage. The on-call teams in these laboratories are expected to meet benchmarks such as door-to-balloon time < 90 minutes. Thus, emergency CABG is required in fewer patients than before. Due to financial and other constraints cardiac surgery team operating on weekends and after working hours face limited resources. However, despite such limitations, these data suggest that the surgical outcomes after regular working hours are comparable after adjusting for the patient factors.

The strengths of the present study include its large sample size and completeness of data. Presence of variables such as estimated mortality allowed us to evaluate patient and procedure-related risk factors as the cause of mortality variation seen in univariable analyses.

On the other hand some limitations are noteworthy. First, great majority of the surgical procedures were performed on weekdays between 7 AM-4 PM and the study reflected a single-center experience. One potential explanation for the former limitation might be related to patient selection during the referral cascade. Indeed, until recently, the emergency department at our facility did not accept ambulances. Thus, patients with cardiac emergencies were first stabilized at other hospitals before being transferred to our medical center. Second, potential external influences such as operator fatigue or limitation of resources, which may adversely influence the mortality of surgery on the weekends or on after hours were not captured in this study. Finally, the study was also limited by its inclusion of mainly male subjects.

## Conclusion

In conclusion, mortality and complications after CABG surgery are higher when the surgery is performed on weekends and after 4 PM in comparison to weekdays and regular working hours, respectively. These timing-related variations in mortality were explained by patient risk factors and urgency of the operation.

## Competing interests

The authors declare that they have no competing interests.

## Authors' contributions

AC and SA constructed the concept and design of the project, performed the analysis, interpreted the data, drafted the manuscript and approved the final manuscript to be published. RC, MK, MA and EOM participated in the analysis and interpretation of data, revised the manuscript critically for important intellectual content and approved the final manuscript to be published.

## Pre-publication history

The pre-publication history for this paper can be accessed here:

http://www.biomedcentral.com/1471-2261/11/63/prepub
